# Mechanism of CuO nano-particles on stimulating production of actinorhodin in *Streptomyces coelicolor* by transcriptional analysis

**DOI:** 10.1038/s41598-019-46833-1

**Published:** 2019-08-02

**Authors:** Xiaomei Liu, Jingchun Tang, Lan Wang, Rutao Liu

**Affiliations:** 1 Key Laboratory of Pollution Processes and Environmental Criteria (Ministry of Education), Tianjin Engineering Research Center of Environmental Diagnosis and Contamination Remediation, College of Environmental Science and Engineering, Nankai University, Tianjin, 300350 China; 2 School of Environmental Science and Engineering, Shandong University, China-America CRC for Environment & Health, 72# Jimo Binhai Road, Qingdao, Shandong 266237 P.R. China

**Keywords:** Bacterial secretion, Soil microbiology, Environmental impact

## Abstract

In this research, antibiotic-producing bacteria, *Streptomyces coelicolor* (*S. coelicolor*) M145, was exposed to copper oxide (CuO) particles to investigate the effects of nano-particles (NPs) on antibiotic production. Results showed that a higher yield of antibiotics was obtained with smaller particle sizes of CuO NPs. When exposed to 10 mg/L of 40 nm CuO NPs, the maximum amount of actinorhodin (ACT) obtained was 2.6 mg/L after 144 h, which was 2.0-fold greater than that of control. However, the process was inhibited when the concentration of CuO NPs was increased to higher than 20 mg/L. Transcriptome analysis showed that all the genes involved in the ACT cluster were significantly up-regulated after exposure to 10 mg/L NPs, which could be the direct cause of the increase of ACT production. Additionally, some genes related to the generation of acetyl-coA were up-regulated. In this way, CuO NPs led to an increase of secondary metabolites. The mechanism related to these changes indicated that nano-particle‒induced ROS and Cu^2+^ played synergetic roles in promoting ACT biosynthesis. This is a first report suggesting that CuO NPs had a significant effect on antibiotic production, which will be helpful in understanding the mechanism of antibiotic production in nature.

## Introduction

*Streptomyces* is a gram-positive bacterium, which is widely relevant as it can currently produce two thirds of antibiotics used in clinical settings^1^. As whole genome sequencing of *Streptomyces coelicolor* (*S. coelicolor*) M145 (henceforth designated as M145), a well-known strain of *Streptomyces*, was carried out in 2002^2^. The most notable finding of the genome sequencing studies was that there are more than 20 gene clusters related to secondary metabolites in this species^3^. Among them, a blue pigmented antibiotic-actinorhodin (ACT) has been widely studied, which belongs to a class of polyketides. More remarkably, ACT is the first antibiotic whose whole biosynthetic gene cluster is cloned^4^. Some studies have reported that ACT could inhibit the growth of gram-positive bacteria^5^. Because of the characteristic blue colour of ACT that enables visual estimation of its production, it has served as an excellent model system for antibiotic regulation^6^.

Antibiotic production of M145 is controlled by many factors, such as transcriptional regulators, proposed coupling of antibiotic synthesis and resistance genes^7^, nutritional and metabolic status^8,9^, and so on. These regulatory mechanisms are easily affected by external environment, such as variations in culture conditions and the addition of different factors to the culture medium^10^. Nanoparticles have been widely reported to have toxic effects on microorganisms; however, there are few reports on whether NPs could affect the secondary metabolites of microbes. Copper oxide nanoparticles (CuO NPs) are a type of common NPs that have been widely used in many fields, such as electronic equipment, superconducting materials, sensors, and novel synthetic antimicrobial agents^11–13^. The increasing usage of CuO NPs inevitably leads to its release in the environment and has an undesirable effect on the environmental organisms. The adverse effect of CuO NPs on microbes, such as *Escherichia coli*, *Bacillus subtilis*, *Streptococcus aureus*^14^, and *Vibrio fischeri*^15^ has drawn much attention in the past few years. Recently, it has been widely accepted that NPs were toxic to various kinds of microbes^16–18^, and many studies have focused on the effects of NPs on growth and development of microorganisms and have illustrated mechanisms related to their toxicity. Our previous study demonstrated that CuO NPs had a significant toxic effect on *Streptomyces coelicolor* M145. However, till date, there are no studies on whether its secondary metabolism, such as production of antibiotics, is also adversely affected by NPs.

The object of this research was to study the effect of CuO NPs on ACT production. Various sizes and concentrations of CuO particles were used during the culture period, and effects of NPs on antibiotic production of M145 were characterized. Transcriptome analyses were applied to evaluate the changes in the genes involved in ACT production at the level of transcription. Furthermore, the mechanisms behind this phenomenon were also investigated by analysing the roles of reactive oxygen species (ROS).

## Materials and Methods

### Materials

CuO particles of 40 nm, 80 nm, 100 nm, and bulk particles (BP) were purchased from Shanghai Macklin Biochemical Company (Shanghai, China); their characterization is reported in our previous study. They were characterized with an average size of 43.5 ± 2.2 nm, 81.5 ± 4.8 nm, 107.0 ± 5.5 nm, and 1329.0 ± 66.9 nm; and an average aggregate size of 998.0 ± 59.6 nm, 1021.0 ± 104.5 nm, 1034.0 ± 88.5 nm, and 1720.0 ± 73 nm, respectively, in an organic rich medium^19^.

### Bacterial cultivation

The model strain, *S. coelicolor* M145, was purchased from China General Microbiological Culture Collection Center (Beijing, China) and was cultured on mannitol soy (MS; soybean flour: 20 g/L, Mannitol: 20 g/L, Agar: 20 g/L) plates for 5–7 days at 30 °C. Later, spores were collected in 20% (v/v) glycerol and preserved at −80 °C^20^. For liquid culture, 1 mL (containing 10^8^ cfu/mL) of spores was inoculated into 100 mL sterilized YBP medium (yeast extract: 2 g/L, beef extract powder: 2 g/L, peptone: 4 g/L, NaCl: 15 g/L, glucose: 10 g/L, MgCl_2_: 1 g/L), and incubated on an orbital shaker at 150 rpm at 30 °C.

### Viability staining and confocal laser scanning microscopy (CLSM)

Cytotoxicity of CuO particles to M145 was determined by LIVE/DEAD Bac-Light bacterial viability kit (L-13152; Invitrogen) after being cultured in YBP medium with various amounts of the particles for 24 h^19^. The cell suspension was centrifuged at 4,000 × *g* for 10 min and washed three times with 0.9% NaCl. Subsequently, a similar volume of Bac-Light solution (SYTO 9: PI = 1:1) was incubated with the cells for 30 min at 30 °C under dark conditions. The fluorescence intensity was measured using a micro-plate reader (Synergy h4, BioTek, USA) with fluorescence wavelengths of green (excitation 485 nm and emission 530 nm) and red (excitation 485 nm and emission 630 nm). The fluorescence images were observed under a confocal laser scanning microscope (LSM880 with Airyscan, Zeiss, German) employing the same wavelengths as those of the microplate reader. Images of all the samples were captured at comparable cell concentrations. The scale of each image was 3.9 mm × 3.9 mm, objective amplification was 10×, and the setting was consistent between the images and exposure conditions.

### Antibiotic extraction and quantification

10 mL culture sample was taken at intervals of 24 h and the sampling continued for one week. 5 mL was used to estimate the dry weights of M145, which were collected on a filter and washed three times with 0.9% NaCl; the filters with bacterial mycelium were then freeze-dried and weighed^21,22^. To determine the ACT concentration, the remaining 5 mL of each sample was centrifuged at 4,000 × *g* for 10 min, supernatant was separated from cells, and an equal volume of 1 M NaOH was added to the supernatant. The samples were left for 1 h at 25 °C before centrifuged at 4,000 × *g* for 5 min, and ACT concentration was determined by measuring the absorbance at 633 nm using a Persee ultraviolet spectrophotometer (T6, Beijing, China)^23^. The antibiotic concentration was calculated from molar extinction coefficients of 25,320 per cm path-length for ACT^24^.

### Culturing M145 on solid medium and scanning electron microscopy (SEM) analysis

YBP solid medium (YBP liquid medium with 20 g/L agar) was prepared with serial concentration of CuO NPs, bacterial spores (10^8^/L) were coated on the solid medium plates, and the bacteria were cultivated for 7 days at 30 °C. From 72 h onwards, pictures of the solid plates were taken every 24 h. The effect of NPs on morphology of the spores was assessed using SEM. After culturing for 96 h, the samples were fixed in 2.5% glutaraldehyde (in 0.1 M phosphate buffer, pH 7.0) overnight, then washed with phosphate buffer (pH 7.0) three times before dehydrating in 30, 50, 70, 80, 90, and 100% methanol successively. Later, the samples were subjected to CO_2_ critical point drying, sputter-coated with gold, and observed using SEM^25^.

### Analysis of Cu^2+^ concentration in the medium and bacterial cells

The assay for the concentration of Cu^2+^ was performed as described in our previous study^19^. Briefly, after shaking for 4 h, the suspensions were centrifuged at 10, 000 × *g* for 20 min^26^, supernatants were filtered twice by 0.22 μm Nylon membranes filters, and Cu^2+^ was estimated using a continuum source atomic absorption spectrometer (contrAA 700, Jena, Germany). For estimating copper (Cu) in the bacterial biomass, M145 was exposed to NPs for 24 h, and then washed three times with 0.9% NaCl. Next, the cells were digested in a microwave digestion system (MDS-15, Sineo, China), filtered through 0.22 μm nylon membrane filters, and the final volume was adjusted to 50 mL with distilled water^27^. Finally, Cu^2+^ was quantified using an inductively coupled plasma with mass spectroscopy (Elan drc-e, Perkin Elmer, USA).

### Intracellular reactive oxygen species (ROS)

An ROS assay kit (Beyotime, China), as described in our previous work^19^, was used for this study; cell permeable 2′,7′-dichlorofluorescein diacetate (DCFH-DA) was used as a fluorescent probe to measure the intracellular ROS concentration. Briefly, after exposure to the probe, cells were centrifuged at 4,000 × *g* and washed three times with 0.9% NaCl. The cells were then suspended in 10 μmol/L DCFH-DA and incubated in the dark at 30 °C for 30 min, followed by washing three times with 0.9% NaCl. The fluorescence intensity was measured using a micro-plate reader with an excitation wavelength of 485 nm and an emission wavelength of 530 nm. The relative ROS level was represented as the fluorescence intensity ratio of the exposure group to the control group, which possessed similar dry masses^28^.

To demonstrate the role of oxidative stress on antibiotic production, 2 mM N-acetylcysteine (NAC), a scavenger of ROS, was added to the YBP medium containing M145 spores, cultured for 1 h before being exposed to pollutants. Meanwhile, M145 was also cultured in other media: one ‒ YBP medium without any added substances as control, the other two with either nano-materials or with Cu^2+^ only. Each treatment was carried out in triplicate. The ROS levels, antibiotic concentration, and expression levels of several genes involved in ACT biosynthesis such as, SCO5071 (*act VI-A*), SCO5072 (*act VI-1*), SCO5082 (*act II-1*), SCO5083 (*act II-2*), SCO5085 (*act II-ORF4*), SCO5086 (*act III*), and SCO5087 (*act I*) were measured.

### Transcriptome sequencing and quantitative real-time RT-PCR

After being cultured for 24 h, M145 exposed to 10 mg/L CuO NPs of 40 nm and to control (comprising only YBP medium) were collected and quickly ground into powder under liquid nitrogen. Next, the M145 powder was added to Trizol (Invitrogen, Carlsbad, USA) and RNA was isolated according to the manufacturer’s instructions. Later, RNA purification, cDNA generation, DNA library construction, gene sequencing, and data analyses were performed by the Gene Denovo Biotechnology Co. (Guangzhou, China) employing Illumina HiSeq^TM^ 2500.

Quantitative real-time RT-PCR (qRT-PCR) was used to verify the accuracy of transcriptome data. The primers used are listed in Table S1, and quantification was performed on a Roche Light Cycler 480 thermal cycler (Roche, Basel, Switzerland) with SYBR Premix Ex Taq (TaKaRa). Reactions were carried out as per the following conditions: 95 °C for 5 min, followed by 40 cycles of 95 °C for 1 min and 60 °C for 1 min. PCR analyses were performed in triplicate for each sample, and *hrdB*^29^, which encoded the major sigma factor of *Streptomyces* was used as an internal control of M145.

### Determination of glucose content

M145 exposed to CuO NPs and control (without NPs) were cultured simultaneously. After 19 h, levels of glucose in the two media were determined by a GOD-POD kit (Leagene, Beijing, China) using the glucose oxidase method according the manufacturer’s instructions. The glucose concentration was determined by measuring the absorbance at 505 nm on an ultraviolet spectrophotometer (T6, Persee, Beijing, China).

### Statistical analysis

Data were expressed as mean ± SD and analysed with IMB SPSS statistics 22 statistical software. Significant differences were assessed by one-way ANOVA with the Student-Newman-Keuls test (S-N-K test), and *p* < 0.05 was considered as statistically significant. Each experiment was performed independently at least three times.

## Results and Discussion

### Effects of different concentrations of CuO NPs particles on antibiotic production and propagation of M145

**Figure 1:**
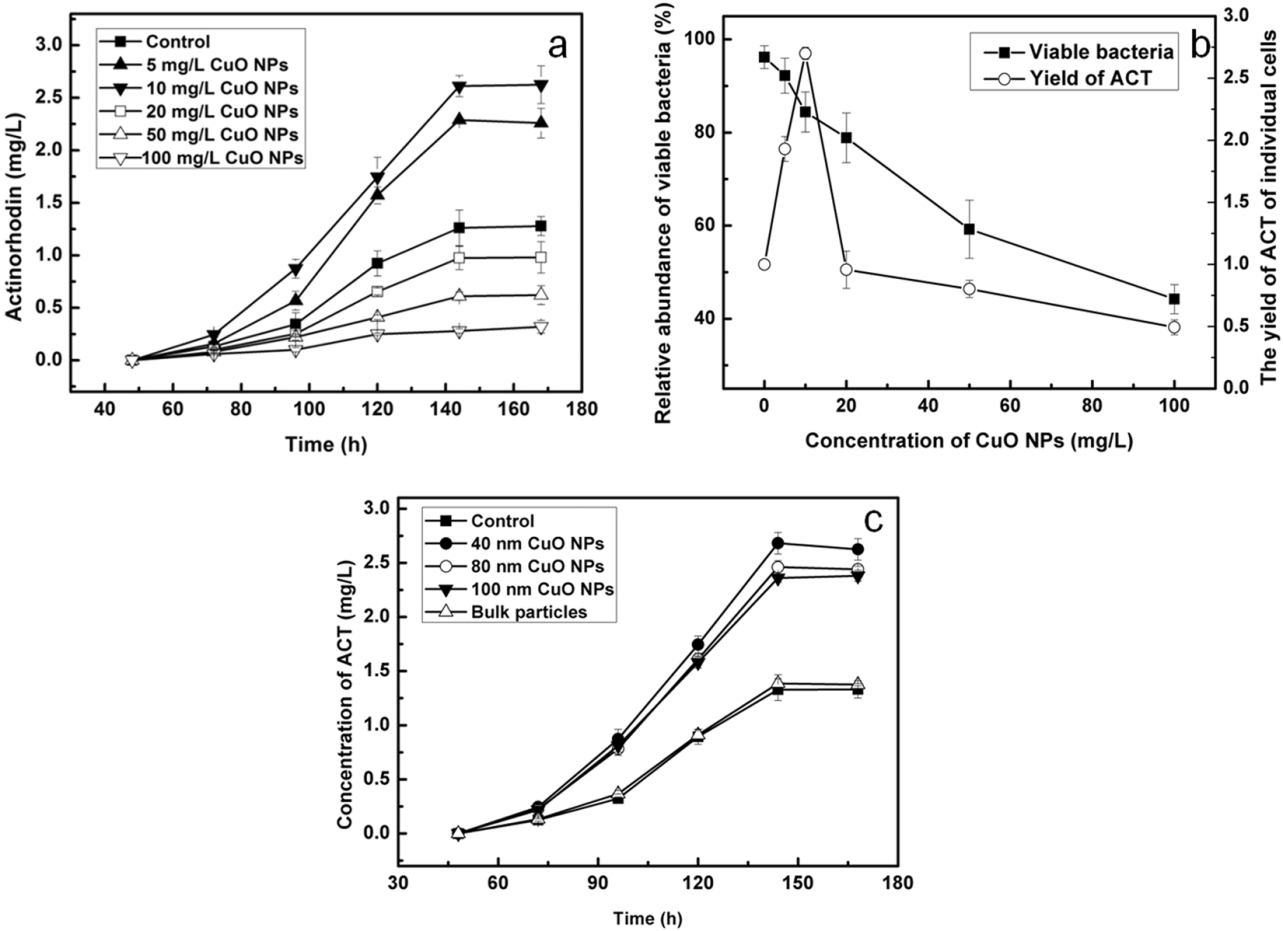
(**a**) Time course of antibiotics production of M145 in the culture medium alone or with addition of different concentrations of CuO NPs of 40 nm; (**b**) Relative abundance of viable bacteria and the ability of producing antibiotics of individual cells treated with different concentration of NPs at 144 h. The left y-axis shows fold changes of viable bacteria of the exposure groups compared to those of control group, which was set as 100%, the right y-axis shows the ability of individual cells to produce antibiotics by the exposure groups compared to that of control group, which was set as one; (**c**) Time course of antibiotic production of M145 in the culture medium alone or with addition of different sizes of CuO NPs at 10 mg/L concentration.

M145 was exposed to different concentrations of CuO NPs particles (0, 5, 10, 20, 50, 100 mg/L) of 40 nm for several days. As shown in Fig. S1, after 60 h, significantly more ACT was produced by the bacteria treated with 10 mg/L CuO NPs than that with other treatments. The yields of ACT became less as the concentration of CuO NPs rose to 20 mg/L or more. The maximum concentration of ACT obtained in the control was 1.3 mg/L at 144 h (Fig. 1a). At lower concentrations, after the addition of CuO NPs, the yield of ACT increased gradually corresponding to the rise in concentrations. When exposed to 10 mg/L CuO NPs, the ACT concentration reached a peak after 144 h and the maximum concentration was 2.6 mg/L, which was 2.0-fold greater than that of control. In this condition, the relative abundance of viable bacteria was 84.4% of control (Fig. 1b). This suggested that although CuO NPs caused some toxicity to bacteria, they could also improve the ability of antibiotic production of individual cells at lower concentrations. When CuO NPs concentration reached 20 mg/L, the ACT yield was 0.76-fold of control at 144 h, while the relative abundance of viable bacteria was 78.9% of control, which meant that with higher concentration of NPs, the ability of antibiotic production of individual cells decreased. When the concentration of CuO NPs was higher than 50 mg/L, the trend was the same as that observed for 20 mg/L.

In order to verify whether nano-particles affected the ACT biosynthesis, M145 were exposed to 10 mg/L CuO particles of varying size: 40 nm, 80 nm, 100 nm, and bulk particles. There were significant differences (*p* < 0.05) in the ACT production between cells exposed to 40 nm and 80 nm (100 nm) CuO NPs, while there was no significant difference (*p* > 0.05) between those exposed to 80 or to 100 nm CuO NPs. As there was no significant difference in the concentration of dissolved copper ions of materials with different sizes (Table 1), it indicated that the effect of different particle sizes led to the difference in ACT production. For particles of 80 nm and 100 nm, there was no obvious difference in particle size; and therefore, the effect on the production of ACT was not significant (Fig. 1c).

**Table 1:** Solubilities of Cu^2+^ in CuO particles of various sizes (40 nm, 80 nm, 100 nm, BPs) and different concentration (5 mg/L, 10 mg/L, 20 mg/L, 50 mg/L and 100 mg/L) in YBP medium after shaking for 24 h.

	5 mg/L	10 mg/L	20 mg/L	50 mg/L	100 mg/L
40 nm	0.98 ± 0.11	2.11 ± 0.32	4.98 ± 0.35	10.23 ± 0.46	25.45 ± 1.24
80 nm	1.03 ± 0.18	2.09 ± 0.21	4.48 ± 0.28	10.56 ± 0.83	24.34 ± 2.51
100 nm	1.12 ± 0.24	2.17 ± 0.22	4.59 ± 0.24	10.25 ± 0.45	23.78 ± 2.90
Bulk particles	0.18 ± 0.05	0.24 ± 0.06	0.34 ± 0.05	0.43 ± 0.07	0.51 ± 0.04

**Figure 2:**
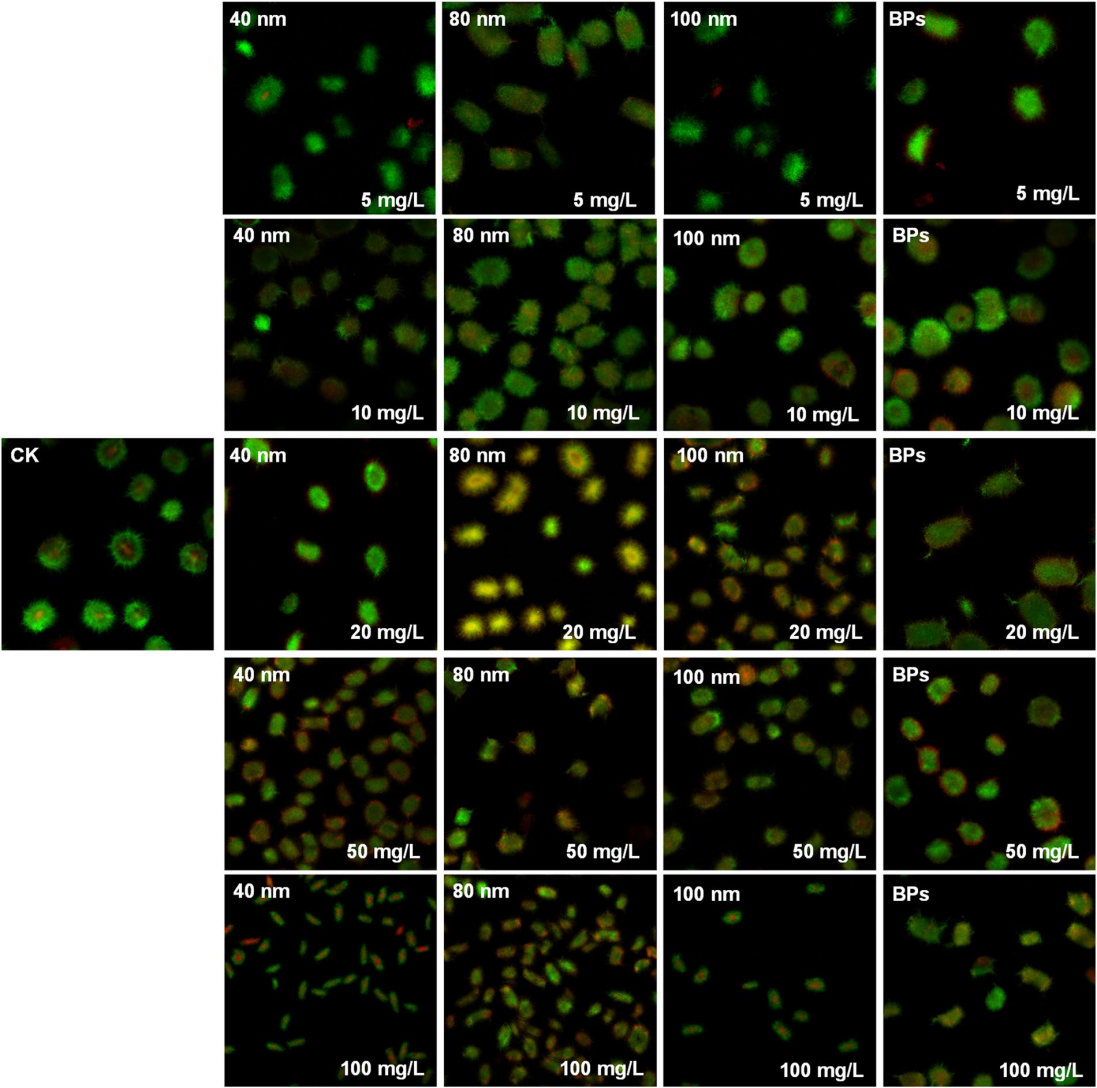
Confocal laser scanning microscope images of M145 exposed to different sizes and concentrations of particles for 24 h (CK: control without any particles, BPs: bulk particles). Cells were stained with SYTO 9 (living cells are stained green) and PI (dead cells stained red) before observing them under a microscope. Images of all the stained samples were captured at comparable cell concentrations. The scale of each image was 3.9 mm × 3.9 mm, objective amplification was 10×, and the setting was consistent among the images.

CLSM were used to examine the size of cells after 24 h (Fig. 2); it was found that the size of pellets became smaller with the treatments of NPs compared to those of control or BPs — the higher the concentration, the smaller the size of cells (Table S2). Combined with our previous finding^19^ with organic-rich medium, CuO existed mainly in the form of ions, and Cu^2+^ entered cells through membranes to cause toxicity; hence, the size of the mycelium did not change although the bacteria were killed. In medium of low nutrition, the solid particles destroyed cell membranes of *Streptomyces*, thus killing the cells, which resulted in smaller pellets. Consequently, it was confirmed that the solid particles had a significant nano-effect on cells in this organic nutrient-poor system. Compared with NPs, the effect of BPs on M145 was much less, both on the growth as well as ACT production (Figs 1c, 2).

**Figure 3:**
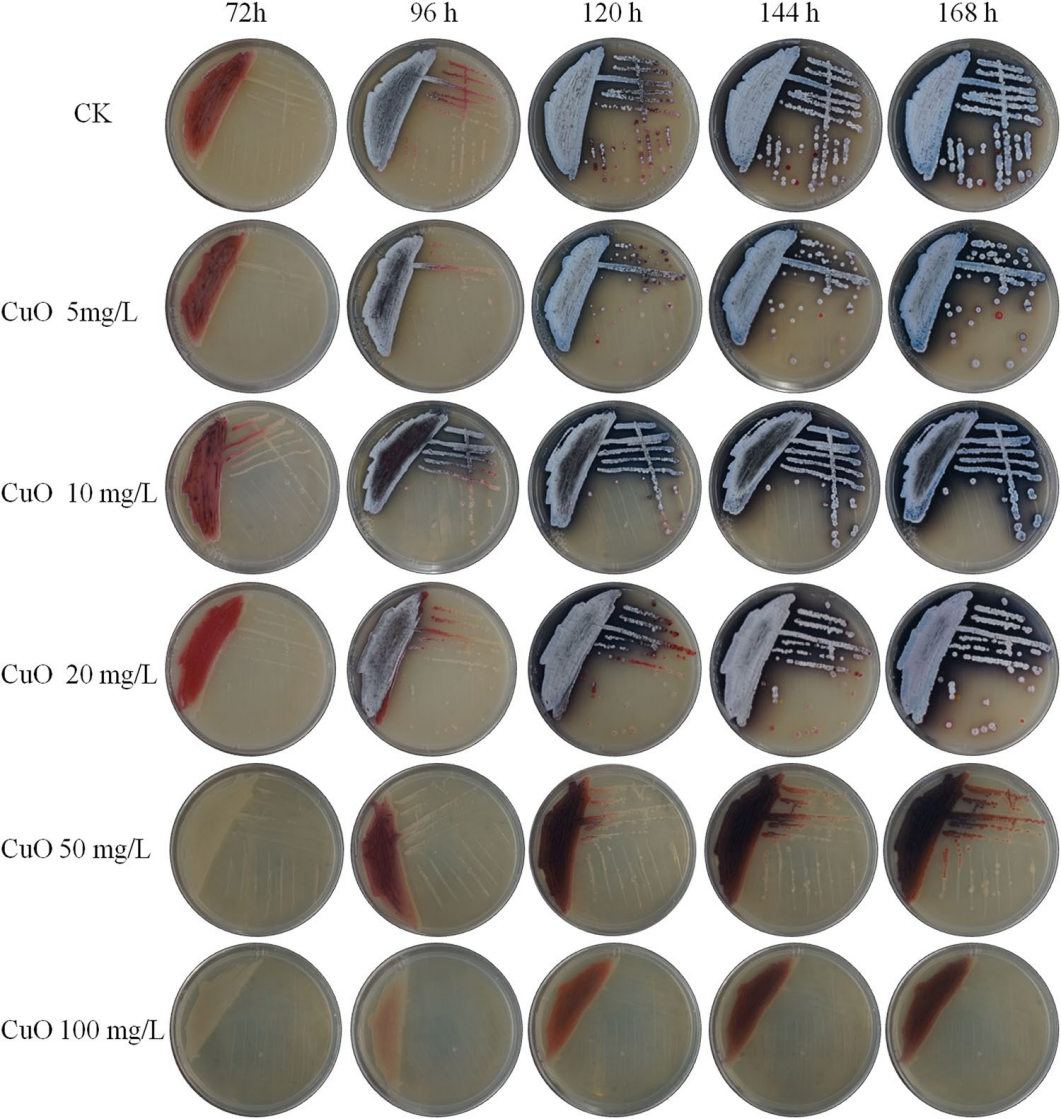
Antibiotic production of M145 cultured on the solid medium with serial concentrations of CuO NPs within 168 h.

To investigate whether CuO NPs had effects on sporulation and production of antibiotic on solid medium, spores were coated on YBP solid medium with serial concentrations of CuO NPs, and photographs were taken from 72 h to 168 h (Fig. 3). It was evident that when the concentration of CuO NPs was 10 mg/L, the ACT yield was higher than that of control. When the concentration was 20 mg/L, the yield was decreased, and the production time was delayed. When the concentration was higher than 50 mg/L, almost no ACT was produced. This result was similar to that of liquid culture. SEM images showed that compared to a complete spore chains exhibited by control bacteria, the aerial hyphae did not differentiate into complete spore chains after 72 h with an addition of 5 mg/L or 10 mg/L CuO NPs (Fig. S2). When the concentration was raised to 50 mg/L or higher, M145 did not produce spores at all, which meant that on solid culture medium, CuO NPs could affect the propagation of M145. As CuO NPs lose the nano effects in solid media, the effects are speculated to be mainly caused by Cu^2+^ ions. The mechanism of action of CuO NPs may be due to the association of Cu^2+^ on bacterial surface followed by its entry into the cells.

In order to detect whether Cu^2+^ enters bacteria, microwave digestion and ICP-MS were performed to measure the content of Cu in cells when exposed to different concentration of NPs. After cultivation for 24 h, the amount of Cu in the cells was the greatest with the addition of 50 mg/L CuO NPs (Table 2). This indicated that a large amount of Cu^2+^ entered into the mycelium from the medium, which had a toxic effect on M145 and led to a reduction in ACT production. In the cells exposed to 5 mg/L or 10 mg/L NPs, there was no significant difference in the Cu content compared with that in control (*p* > 0.05). Thus, it can be inferred that in this situation, only trace amounts of Cu entered the cells, which was not enough to cause cytotoxicity, but other factors, such as nano-particle effects might have played a role in enhancing antibiotic biosynthesis.

**Table 2:** Intracellular copper content of M145 after treated with different concentrations of NPs for 48 h (n = 3).

NPs	Control	5 mg/L	10 mg/L	20 mg/L	50 mg/L	100 mg/L
Cu^2+^ (µg/g)	9.7 ± 0.5	9.9 ± 0.4	10.1 ± 0.7	17.1 ± 1.1	30.6 ± 2.0	24.7 ± 1.5

### ROS Effects on secondary metabolism of M145

**Figure 4:**
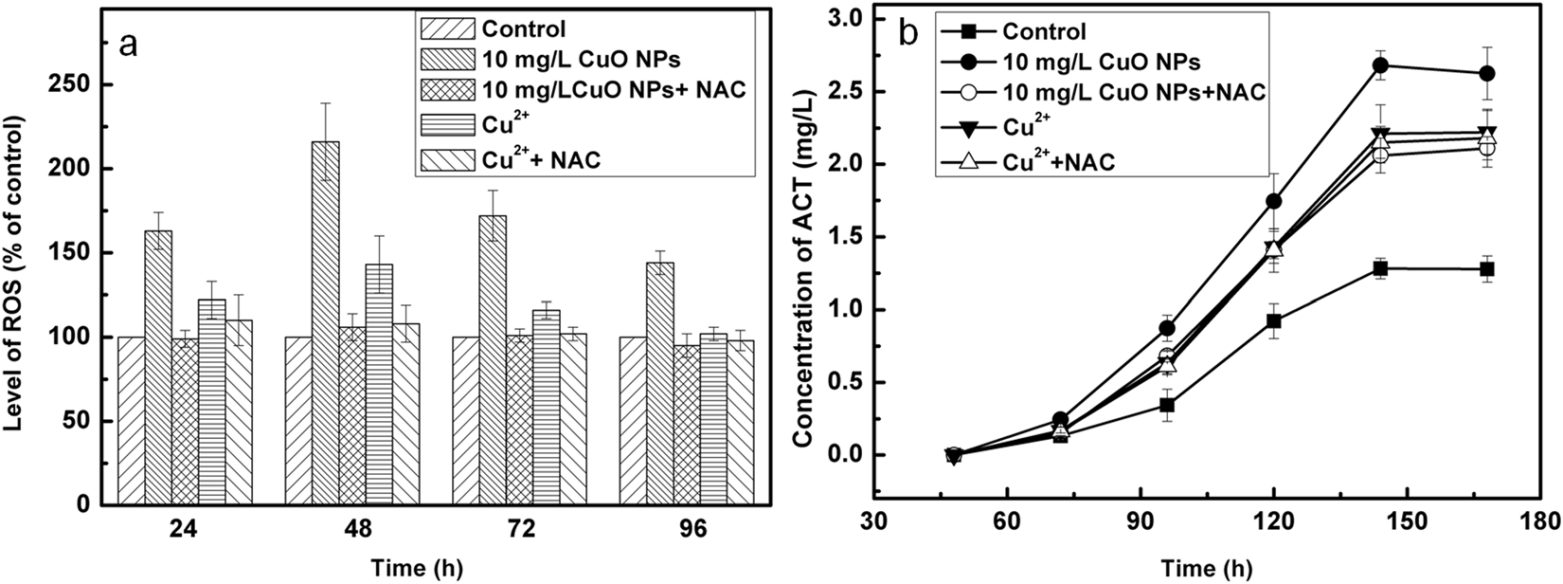
Time course of fermentation process by M145 cultivated in YBP medium with 10 mg/L CuO NPs, 2.1 mg/L copper ions (Cu^+^), 10 mg/L CuO NPs and 2 mM NAC (N-acetylcysteine, a scavenger of ROS), 2.1 mg/L Cu^+^ and 2 mM NAC, or YBP medium without any added substances as control. (**a**) Time course of ROS level of M145 after different treatments, y-axis shows fold changes of ROS level of different experimental groups compared to the level of control at each time point, the latter was set to 100%; (**b**) Time course of actinorhodin (ACT) levels with different treatments.

It remained to be seen how solid particles work on the process of ACT biosynthesis. Results of recent studies indicated that oxidative stress and dissolved ions played important roles in toxicities of metal NPs^30–32^. It was observed that the content of Cu^2+^ in YBP medium was 2.1 mg/L when the concentration of CuO NPs was 10 mg/L, and at this concentration the toxicity was not obvious^19^. When 2.1 mg/L Cu^2+^ alone was added to the medium, the maximum ACT concentration was 2.2 mg/L after the bacteria were cultured for 144 h, which was 1.7-fold greater than that of control, but lower than an exposure to 10 mg/L NPs. To clarify the role of ROS in this process, ROS eliminating agent N-acetylcysteine (NAC) was added 1 h before treatment with 10 mg/L NPs and 2.1 mg/L Cu^2+^. After culturing the cells for 48 h, concentrations of ROS in M145 reached maximum amounts when they were exposed to either NPs or Cu^2+^, the former being much higher than the latter. After the addition of NAC, whether related to NPs or Cu^2+^, the concentrations of ROS reduced to those equal to controls, which indicated that effects of ROS were substantially eliminated (Fig. 4a). Compared to expose to NPs alone, the addition of NAC led to a reduction of ACT production in cells, and it was not significantly different than in the cells exposed to Cu^2+^ (*p* > 0.05). This indicated that ROS played a significant role in promoting ACT production. Compared to exposure to Cu^2+^ alone, the yield of ACT did not change significantly (*p* > 0.05) with the addition or in absence of NAC (Fig. 4b). This indicated that the effect of ROS mainly originated from NPs, and those caused by Cu^2+^ were minimal, so as to be ignored.

### Differential expression of genes involved in actinorhodin biosynthesis after exposure to CuO NPs

**Figure 5:**
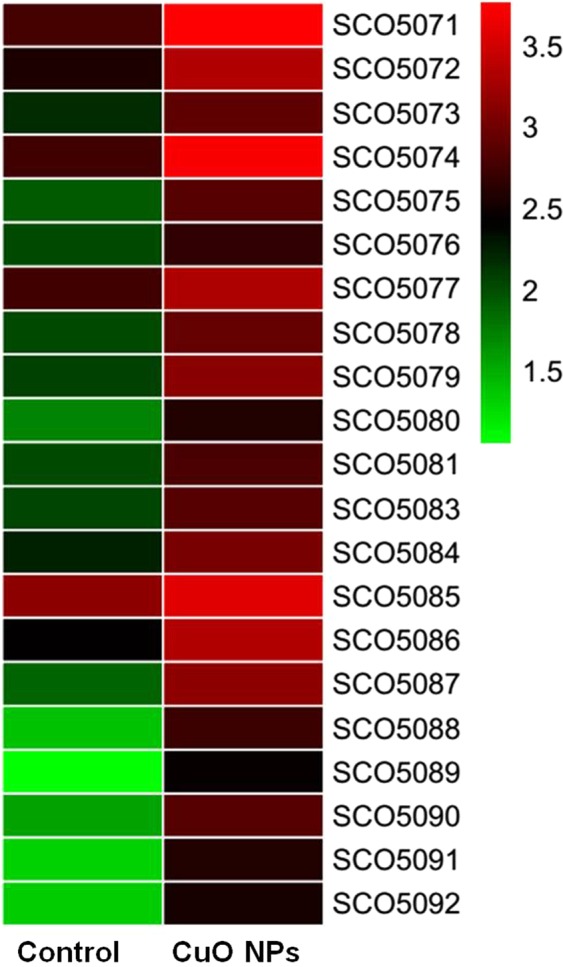
Profiles of relative expressions of each gene after exposure or without exposure to CuO NPs for 24 h. CuO NPs: exposed to 10 mg/L CuO NPs (40 nm).

Transcriptome sequencing was carried out to investigate the response of M145 to 10 mg/L CuO NPs (40 nm). Gene expression was analysed after exposure to the particles for 24 h. Genes involved in ACT biosynthesis reside in a single cluster (SCO5071 to SCO5092)^2^. Results showed that after exposure to 10 mg/L NPs, all the genes involved in ACT cluster were significantly up-regulated, which could be a direct cause of the increase of ACT production. Because ACT is a type of secretory antibiotic, specific transport mechanisms were needed for its transfer to the extracellular domain. To meet the requirement, M145 also encoded three putative export pumps: actII-ORF2, actII-ORF3, and actVA-ORF1^33^. Transcription of *actVA-ORF1* (SCO5076), which can export more antibiotics to reduce cytotoxicity, was significantly increased after exposure to NPs (Fig. 5).

The key substrate for synthesis of ACT was acetyl-coA, a starter in the polyketide pathway; eight acetyl-coA, under the action of ActI-1, 2, 3, can synthesize the skeleton of ACT^5^. Consequently, the increase of acetyl-coA leads to the stimulation of antibiotic biosynthesis. Acetyl-coA is mainly produced by the degradation of several amino acids and carbon metabolism. It was found that 17 genes involved in valine, leucine, and isoleucine degradation were dramatically changed, 15 of which were up-regulated (Table S3) and several genes involved in carbon metabolism were also up-regulated (Table S4), resulting in the production of more acetyl-coA. In order to improve the ability of carbon metabolism, cells need to take more sugars from their external milieu. In our study, transcriptome analysis showed that the expression of 23 genes of ABC transporters involved in the transport of sugars were up-regulated (Table S5), which promoted the absorption and metabolism of sugars in the cells.

**Figure 6:**
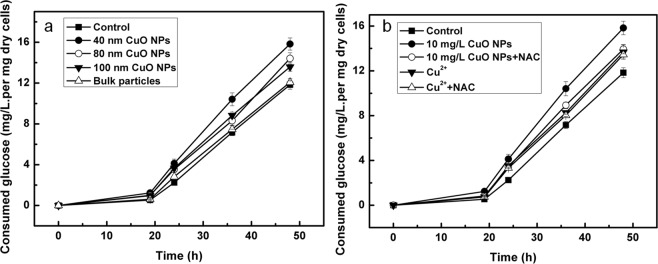
Time course of glucose uptake of M145 grown in culture medium alone or exposed to different pollutants for 48 h. (**a**) Time course of glucose uptake of M145 in culture medium alone or exposed to different size CuO particles for 48 h; (**b**) Time course of glucose uptake of M145 cultivated in YBP medium with 10 mg/L CuO NPs (40 nm), 2.1 mg/L copper ion, 10 mg/L CuO NPs and 2 mM NAC (N-acetylcysteine, a scavenger of ROS), 2.1 mg/L Cu^2+^ and 2 mM NAC, or YBP medium without any added substances as control.

Furthermore, glucose consumption was also estimated as an example to explain the mechanism of NPs in stimulating carbon metabolism (Fig. 6). When different sizes of CuO NPs of 10 mg/L were added to the medium, glucose uptake changed dramatically compared to that of control. Moreover, we demonstrated that all the three kinds of NPs led to an acceleration of glucose uptake. The glucose uptake (15.8 mg/L per mg dry cells) of cells exposed to 40 nm particles at 48 h was 33.9% higher than that of control (11.8 mg/L per mg dry cells). Regarding BPs, there were no significant difference in the glucose uptake in the treatment groups compared to that in control (*p* > 0.05). However, there were significant differences observed (*p* < 0.05) in cells exposed to 40 nm and 80 nm (100 nm) NPs, while there was no significant difference (*p* > 0.05) between those exposed to 80 and 100 nm NPs, which was consistent with the propensity of the cells to produce antibiotics (Fig. 6a).

Similar to NPs, the addition of Cu^2+^ could also lead to an acceleration of glucose uptake. The glucose uptake (13.7 mg/L per mg dry cells) of the culture medium with added Cu^2+^ was a little higher than that of control, but lower than that exposed to 10 mg/L NPs (Fig. 6b). From the results obtained after the addition of NAC to eliminate the effect of ROS, combined with the previous analysis, it can be concluded that the nano-particle-induced ROS along with Cu^2+^ could affect glucose uptake, leading to the production of more acetyl-coA, thereby affecting antibiotic biosynthesis.

To assess the effects of ROS on gene expression, seven genes involved in ACT biosynthesis were chosen for analysis. Compared to cells exposed only to NPs (10 mg/L, 40 nm), the expression level of these genes decreased significantly after eliminating intracellular ROS (Fig. S3a), indicating that ROS played an important role in improving the expression of ACT genes. As pathway-specific regulators are generally considered to have a most direct effect on antibiotic production via transcriptional activation of the relevant biosynthetic genes, the pathway-specific regulatory protein was revealed to be ActII-ORF4 in the biosynthesis of ACT^34,35^. Factors that influence the production of ACT are mostly affected by regulation of transcription or translation of *actII-ORF4* (SCO5085)^36^. In this study, to assess the roles of Cu^2+^, *actII-ORF4* was chosen, and it was found that there was no significant difference in gene expression between Cu^2+^ treatment and ROS-free CuO NPs treatment at different time points. Additionally, it was revealed that whether ROS was eliminated or not, no significant effect was seen on the gene expression with Cu^2+^ treatment (Fig. S3b). This suggested that nano-particle-induced ROS and Cu^2+^ both affected the expression of antibiotic genes.

It was also found that the expression of several genes related to the two–component system (TCS) was changed dramatically after exposure to CuO NPs (Table S6). TCS is a type of global regulator that is a predominant signal transduction system employed by bacteria to monitor and respond to changing environments^36^. Typical TCS consists of a sensor kinase and a response regulator such as *kdpA*/*kdpB* (SCO3717/3718) and *mprB*/*mprA* (SCO4155/4156), which sense external changes of bacteria after NPs are added with sensor kinases and transfer the signals to cells. Genes, such as *tctC* (SCO1138), *tctB* (SCO1139), *tctA* (SCO1140), are related to tricarboxylate transport. In this study, after addition of CuO NPs, the expression of genes involved in the tricarboxylic acid cycle was improved (Table S3); it is likely that TCS may have perceived this change and sent a signal to other components of the cell, causing a series of changes. Similarly, *atoB* (SCO5399) could transmit changes of acetoacetate, and *uhpB* (SCO6424), a kind of histidine kinase can transfer the changes of glucose-6-phosphate to other components of cells. These genes could have activated the expression of antibiotic genes and regulated the production of ACT. These results suggest that CuO NPs increased the production of acetyl-coA by promoting primary metabolic pathways, which ultimately led to an increase of secondary metabolites.

To validate transcriptome data, seven genes from the ACT pathway were quantified by qRT-PCR; *hrdB* was used as the internal control of M145. Fold changes obtained from the results of qRT-PCR were consistent with those of transcriptome analysis (Fig. S4).

## Conclusion

In this research, effects of NPs on the production of antibiotics were studied using the strain, *S. coelicolor* M145. It was observed that low concentration of CuO NPs could increase the production of antibiotics, smaller the particle size, higher the amount of antibiotic produced; however, high concentration of NPs inhibited this process. Compared with NPs, the effect of BPs on growth and biosynthesis of ACT by M145 was much less. From these results, we concluded that both nano-particle-induced ROS and Cu^2+^ together play crucial roles in responding to the effects of NPs on antibiotic production, showing an effect of 35% and 65%, respectively. The addition of CuO NPs to the fermentation medium resulted in the up-regulation of several genes related to acetyl-coA (a key substrate for the synthesis of ACT), such as 15 genes involved in valine, leucine and isoleucine degradation and several genes involved in carbon metabolism. In addition, the expression of some TCS genes also changed, which could have activated the expression of antibiotic genes. In this way, CuO NPs led to an increase of secondary metabolites. Our studies reveal that NPs play an important role in stimulating the production of antibiotics, which is helpful in understanding the mechanism of antibiotics production in nature. In addition, the result provided important implications for exploring usage of other NPs in bio-medical applications or regulation of antibiotics in nature.

## Supplementary information

Mechanism of CuO nano-particles on stimulating production of actinorhodin in Streptomyces coelicolor by transcriptional analysis

## Data Availability

All data generated or analysed during this study are included in this published article and its Supplementary Information Files.
