# Supplementary material for: Mechanism of CuO nano-particles on stimulating production of actinorhodin in Streptomyces coelicolor by transcriptional analysis
Source: Sci Rep. 2019 Aug 2;9:11253. doi: 10.1038/s41598-019-46833-1 (PMC6677739; doi:10.1038/s41598-019-46833-1)
Supplement: Supplementary file 1 — Mechanism of CuO nano-particles on stimulating production of actinorhodin in Streptomyces coelicolor by transcriptional analysis [file 41598_2019_46833_MOESM1_ESM.docx]

**Mechanism of CuO nano-particles‎ on stimulating production of actinorhodin in *Streptomyces coelicolor* by transcriptional analysis**

Xiaomei Liu^a^, Jingchun Tang*^a^, Lan Wang^a^, Rutao Liu^b^

^a^Key Laboratory of Pollution Processes and Environmental Criteria (Ministry of Education), Tianjin Engineering Research Center of Environmental Diagnosis and Contamination Remediation, College of Environmental Science and Engineering, Nankai University, Tianjin 300350, China.

^b^School of Environmental Science and Engineering, Shandong University, China-America CRC for Environment & Health, 72# Jimo Binhai Road, Qingdao, Shandong 266237, PR China

*Corresponding author E-mail: [tangjch@nankai.edu.cn](mailto:tangjch@nankai.edu.cn) <Tel:13682055616>

Table S1 List of primers used for quantitative RT-PCR

| Gene | Name |  | Primer sequence |
| --- | --- | --- | --- |
| SCO5071 | *actⅥ-A* | F | CGGCCACCAGATGCAGAA |
|  |  | R | TTGCGGGCGTCGAACTTGC |
| SCO5072 | *actⅥ-1* | F | CACGCCCTCGTGCTGTCCT |
|  |  | R | CGATGTGCGGTGGGTTGAA |
| SCO5082 | *actⅡ-1* | F | CGGTCGCTGCGGAGGATGTT |
|  |  | R | TAGGCGGCGAGTTCGTCGTG |
| SCO5083 | *actⅡ-2* | F | GGCTGGGCGACATCTACGG |
|  |  | R | TGGGCGGGAACATTTGCT |
| SCO5085 | *actⅡ-ORF4* | F | CCTGGTGCTGCTGCTCCTCA |
|  |  | R | ATTCCCGGTCGTCGCTCGTC |
| SCO5086 | *actⅢ* | F | GAAGAAGGACTGCGGACGACG |
|  |  | R | GACCGTAACGCTCGACCACC |
| SCO5087 | *actⅠ* | F | GACGATGACGACGACCACCGGACGAA |
|  |  | R | CCCGAGGTGAGCAGTTCCCAGAA |
| SCO5820 | *hrdB* | F | GGGCTACAAGTTCTCCACG |
|  |  | R | AGGTCCTGGAGCATCTGGC |

Table S2. The diameter of mycelia pellets treated with different size and different concentration of NPs for 24 h.

|  | 5 mg/L  (mm) | 10 mg/L  (mm) | 20 mg/L  (mm) | 50 mg/L  (mm) | 100 mg/L  (mm) |
| --- | --- | --- | --- | --- | --- |
| 40 nm | 0.28±0.04 | 0.26±0.01 | 0.23±0.01 | 0.16±0.01 | 0.08±0.01 |
| 80 nm | 0.33±0.02 | 0.30±0.02 | 0.26±0.02 | 0.20±0.02 | 0.12±0.01 |
| 100 nm | 0.29±0.04 | 0.32±0.02 | 0.30±0.03 | 0.19±0.02 | 0.14±0.01 |
| Bulk particles | 0.33±0.04 | 0.34±0.03 | 0.30±0.02 | 0.26±0.06 | 0.22±0.04 |

Control without any treatment was 0.35±0.05mm (n=10)

Table S3: Genes involved in valine, leucine and isoleucine degradation that were dynamically changed.

| Gene ID | Gene name | Biological role | Fold change (log_2_C) |
| --- | --- | --- | --- |
| SCO1324 | *fadA* | acetyl-CoA acetyltransferase | -3.0 |
| SCO1706 | *sapD* | aldehyde dehydrogenase | 1.2 |
| SCO2774 | *acaDL* | acyl-CoA dehydrogenase | 1.1 |
| SCO2778 | *hmgCL* | hydroxymethylglutaryl-CoA lyase | 1.3 |
| SCO2779 | *acdC* | acyl-CoA dehydrogenase | 1.0 |
| SCO4381 | *mccA* | acetyl-CoA carboxylase | 1.2 |
| SCO4913 | *aldH5* | aldehyde dehydrogenase | 1.4 |
| SCO4921 | *bccA* | biotin carboxyl carrier protein | 1.2 |
| SCO5398 | *mce* | methylmalonyl-CoA epimerase | -1.2 |
| SCO5399 | *fadA4* | acetyl-CoA acetyltransferase | 1.0 |
| SCO5523 | *ilvE* | branched-chain amino acid aminotransferase | 1.1 |
| SCO5676 | *gabT* | 4-aminobutyrate aminotransferase | 1.3 |
| SCO5679 | *ladH* | aldehyde dehydrogenase | 1.2 |
| SCO6271 | *bccA* | biotin carboxyl carrier protein | 1.1 |
| SCO6701 | *paaJ* | acetyl-CoA acetyltransferase | 1.0 |
| SCO6703 | *scoA* | 3-oxoadipate CoA-transferase subunit A | 1.8 |
| SCO6967 | *pcaF* | beta-ketoadipyl-CoA thiolase | 1.2 |

Table S4: Genes involved in carbon metabolism that were up-regulated.

| Gene ID | Gene name | Biological role | Fold change (log_2_C) |
| --- | --- | --- | --- |
| SCO0379 | *catA* | Catalase | 1.6 |
| SCO0922 | *sdhB* | succinate dehydrogenase | 1.4 |
| SCO0923 | *sdhA* | succinate dehydrogenase flavoprotein subunit | 1.7 |
| SCO0924 | *---* | succinate dehydrogenase | 1.3 |
| SCO1378 | *gcsP* | glycine dehydrogenase | 1.8 |
| SCO2183 | *odp1* | 2-oxoacid dehydrogenase subunit E1 | 1.7 |
| SCO2494 | *ppdK* | pyruvate phosphate dikinase | 1.7 |
| SCO2774 | *acaDL* | acyl-CoA dehydrogenase | 1.1 |
| SCO3817 | *bkdA* | branched-chain alpha-keto acid dehydrogenase E1 subunit alpha, partial | 1.3 |
| SCO4381 | *mccA* | acetyl-CoA carboxylase subunit alpha | 1.2 |
| SCO4827 | *mdh* | malate dehydrogenase | 1.3 |
| SCO4921 | *bccA* | biotin carboxyl carrier protein | 1.2 |
| SCO5399 | *fadA4* | acetyl-CoA acetyltransferase | 1.0 |
| SCO5469 | *sdhL* | L-serine dehydratase | 1.2 |
| SCO5470 | *glyA* | serine hydroxymethyltransferase | 1.5 |
| SCO5999 | *acnA* | aconitate hydratase | 1.29 |
| SCO6204 | *catA* | catalase | 1.6 |
| SCO6243 | *masY* | malate synthase | 1.4 |
| SCO6270 | *korA* | 2-oxoglutarate ferredoxin oxidoreductase subunit alpha | 1.0 |
| SCO6271 | *bccA* | biotin carboxyl carrier protein | 1.1 |
| SCO6659 | *g6pI1* | glucose-6-phosphate isomerase | 1.1 |
| SCO6661 | *g6pD2* | glucose-6-phosphate 1-dehydrogenase | 1.1 |
| SCO6662 | *tal1* | transaldolase | 1.2 |
| SCO6663 | *tkt* | transketolase | 1.7 |
| SCO7000 | *idh* | isocitrate dehydrogenase | 1.2 |
| SCO7040 | *gap2* | glyceraldehyde-3-phosphate dehydrogenase | 1.7 |
| SCO7638 | *eno2* | enolase | 1.2 |

Table S5: Genes related to ABC transporters that were up-regulated.

| Gene ID | Gene name | Biological role  (transporters) | Fold change (log_2_C) |
| --- | --- | --- | --- |
| SCO0353 | *msmF* | Raffinose, Stachyose,  Melibiose | 1.6 |
| SCO0354 | *msmG* |  | 2.5 |
| SCO0449 | *rhaS* | Rhamnose | 1.4 |
| SCO0881 | *rhaT* |  | 2.5 |
| SCO1898 | *somE* | Sorbitol  Mannitol | 3.9 |
| SCO1899 | *somF* |  | 4.0 |
| SCO1900 | *somG* |  | 3.8 |
| SCO2229 | *ganQ* | Galactose oligomer | 1.7 |
| SCO2230 | *ganP* |  | 1.6 |
| SCO2795 | *cebE* | Cellobiose | 2.2 |
| SCO2796 | *cebF* |  | 1.3 |
| SCO2797 | *cebG* |  | 2.0 |
| SCO4240 | *malK* | Maltose, α-Glucoside | 1.9 |
| SCO5233 | *dasB* | Chitobiose | 11.4 |
| SCO6005 | *ngcE* | N-Acetylglucosamine | 1.1 |
| SCO6007 | *ngcG* |  | 1.2 |
| SCO6009 | *xylF* | D-Xylose | 1.3 |
| SCO6010 | *xylG* |  | 1.7 |
| SCO6011 | *xylH* |  | 1.5 |
| SCO6567 | *rbsA* | Ribose | 2.6 |
| SCO6568 | *rbsC* |  | 2.5 |
| SCO6569 | *rbsB* |  | 1.7 |
| SCO7028 | *bxlE* | Xylobiose | 1.1 |

Table S6: Genes involved in two-component system that were dynamically changed.

| Gene ID | Gene name | Biological role | Fold change (log_2_C) |
| --- | --- | --- | --- |
| SCO0219 | *narI* | nitrate reductase subunit gamma | 1.1 |
| SCO1138 | *tctC* | C4-dicarboxylate ABC transporter substrate-binding protein | 5.1 |
| SCO1139 | *tctB* | membrane protein | 3.6 |
| SCO1140 | *tctA* | tripartite tricarboxylate transporter TctA | 3.1 |
| SCO1396 | *vanX* | D-alanyl-D-alanine dipeptidase | 1.7 |
| SCO3717 | *kdpA* | two component sensor kinase | 1.2 |
| SCO3718 | *kdpB* | two-component system response regulator | 1.3 |
| SCO4155 | *mprB* | two component sensor kinase | 1.6 |
| SCO4156 | *mprA* | two-component system response regulator | 2.0 |
| SCO5399 | *atoB* | acetyl-CoA acetyltransferase | 1.0 |
| SCO5584 | *glnB* | nitrogen regulatory protein | -2.3 |
| SCO5585 | *glnD* | uridylyltransferase | -1.5 |
| SCO6354 | *mprA* | Serine protease | 1.3 |
| SCO6424 | *uhpB* | histidine kinase | 1.6 |
| SCO6962 | *glnA* | glutamine synthetase | -1.5 |


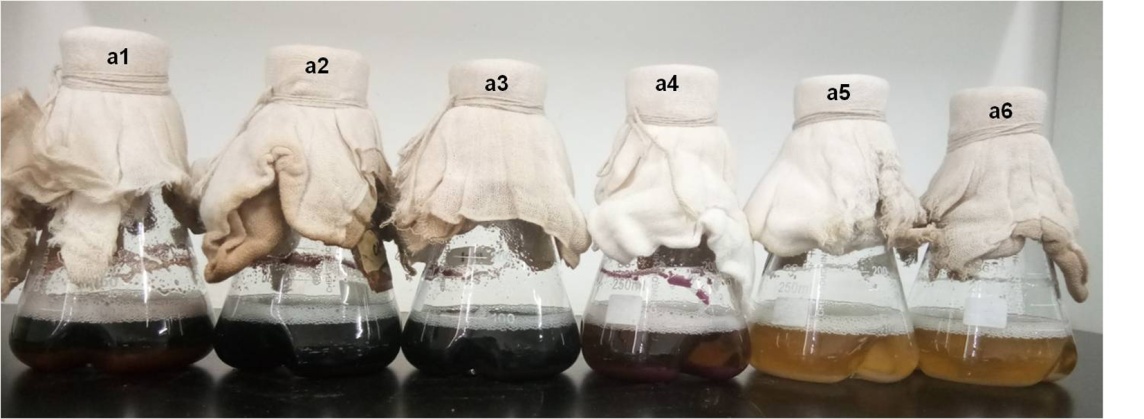


a

Figure S1: Antibiotics production of M145 in the pure culture and interacted with CuO NPs after 60 hours: a1: control; a2: treated with 5 mg/L CuO NPs; a3: treated with 10 mg/L CuO NPs; a4: treated with 20 mg/L CuO NPs;. a5: treated with 50 mg/L CuO NPs; a6: treated with 100 mg/L CuO NPs.


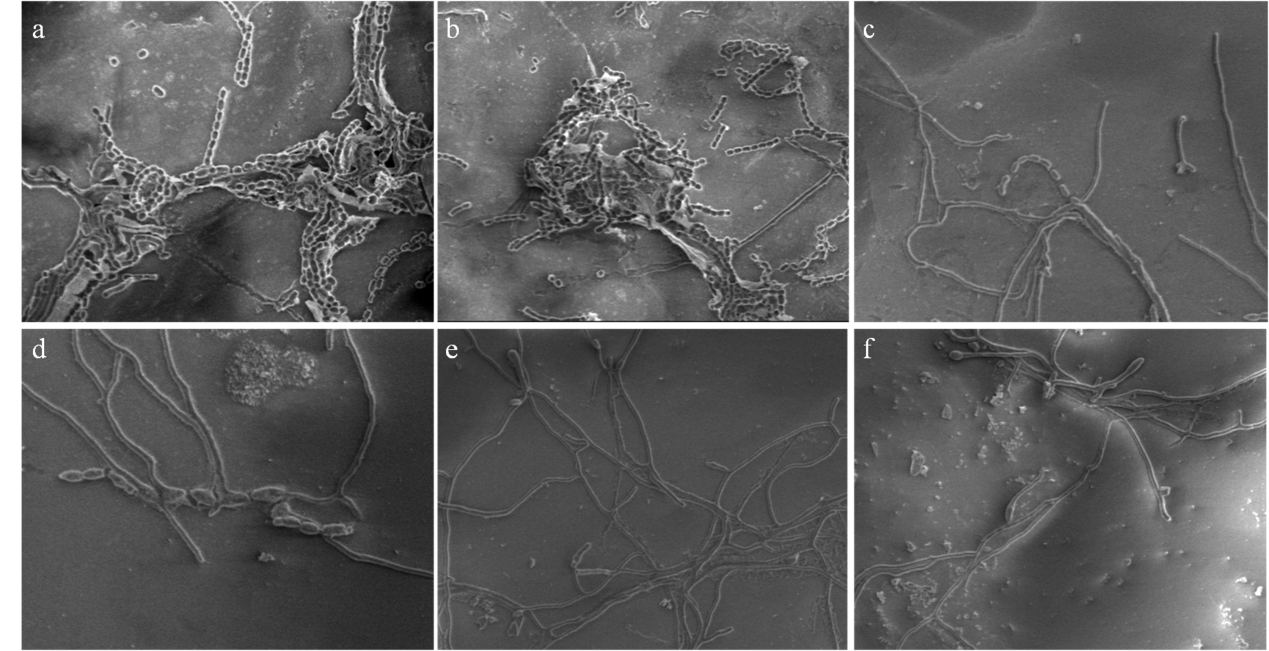


Figure S2: SEM images of cultured for four days. a: control without any CuO NPs; b: with an addition of 5 mg/L CuO NPs; c: with an addition of 10 mg/L CuO NPs; d: with an addition of 20 mg/L CuO NPs; e: with an addition of 50 mg/L CuO NPs; d:with an addition of 100 mg/L CuO NPs.

**
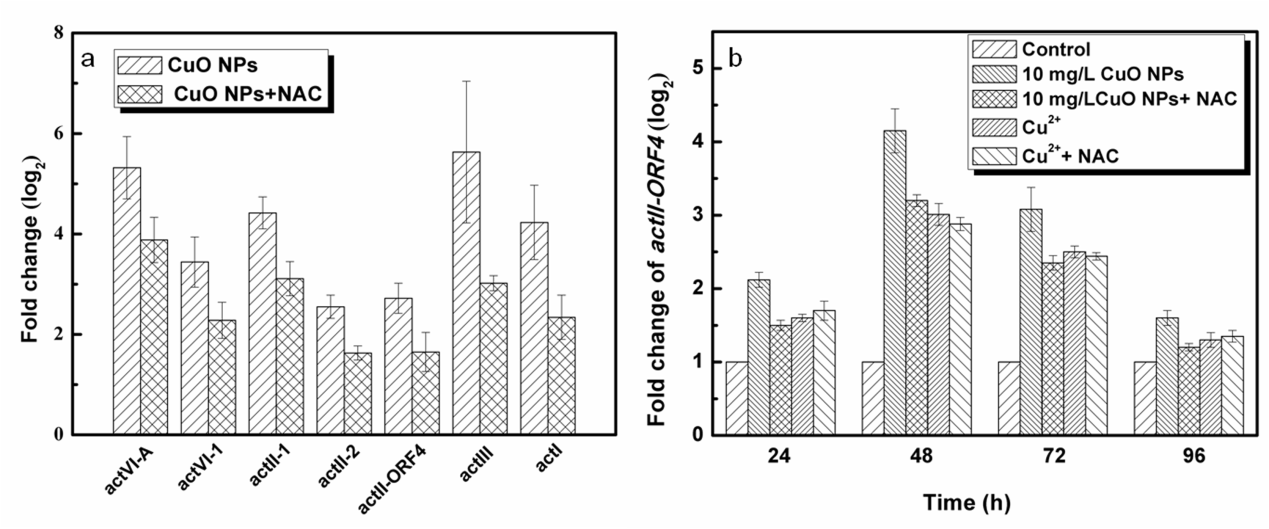
**

Figure S3: ROS effects the expression of several genes involved in ACT biosynthesis. a: Gene expression before and after the elimination of ROS; b: Time course of *actII-ORF4* gene expression after cultivated in YBP medium with 10 mg/L CuO NPs, 2.1 mg/L copper ion, 10 mg/L CuO NPs and 2 mM NAC (N-acetylcysteine, a scavenger of ROS), 2.1 mg/L copper ion and 2 mM NAC respectively or pure YBP medium without any materials as control. The y-axis shows the fold change in the expression levels in the two experimental groups compared with the control level at each time point, which was set to one.

Figure S4: Validation of transcriptome data by quantitative RT-PCR. The fold changes of 7 genes involved in ACT gene cluster were determined by quantitative RT-PCR and the transcriptome analysis.
